# Correction to: ‘Visualization for epidemiological modelling: challenges, solutions, reflections and recommendations’ (2022) by Dykes *et al.*

**DOI:** 10.1098/rsta.2022.0296

**Published:** 2022-10-31

**Authors:** Jason Dykes, Alfie Abdul-Rahman, Daniel Archambault, Benjamin Bach, Rita Borgo, Min Chen, Jessica Enright, Hui Fang, Elif E. Firat, Euan Freeman, Tuna Gönen, Claire Harris, Radu Jianu, Nigel W. John, Saiful Khan, Andrew Lahiff, Robert S. Laramee, Louise Matthews, Sibylle Mohr, Phong H. Nguyen, Alma A. M. Rahat, Richard Reeve, Panagiotis D. Ritsos, Jonathan C. Roberts, Aidan Slingsby, Ben Swallow, Thomas Torsney-Weir, Cagatay Turkay, Robert Turner, Franck P. Vidal, Qiru Wang, Jo Wood, Kai Xu

*Phil. Trans. R. Soc. A* **380**, 20210299. (Published online 15 August 2022). (https://doi.org/10.1098/rsta.2021.0299)

In the original version of this article, references 113–120, 123–140 and 143 were incorrectly numbered.

This has been corrected on the publisher's website.

